# Gender Bias in ADHD Assessment: Are Male Partners Underestimating Female Symptoms on CAARS?

**DOI:** 10.1192/j.eurpsy.2025.428

**Published:** 2025-08-26

**Authors:** L. Moody, W. Mohamed

**Affiliations:** 1 Mental Health and Wellbeing Services, Shrewsbury, United Kingdom

## Abstract

**Introduction:**

Social support can significantly influence mental health help-seeking (Rickwood et al., 2005). At MHWS, many patients are older and receiving late diagnoses for ADHD, often after experiencing lifelong difficulties. Research shows that women’s mental health issues are frequently overlooked due to gender biases in healthcare (Kuehner, 2017), with men often underestimating the severity of their partners’ mental health challenges (O’Neil, 2008; Cummings & Davies, 2002). This study explores whether similar patterns of underrepresentation occur in ADHD diagnoses, particularly in relation to observer gender and relationship to the patient.

**Objectives:**

The study aimed to explore differences in ADHD assessment scores between patients and their observers based on the observer’s gender and relationship to the patient. Additionally, it sought to determine whether the underrepresentation of ADHD symptoms differs based on these variables.

**Methods:**

A cross-sectional comparative study was conducted involving 196 patients (123 females, 73 males) and their observers (135 females, 61 males) using the Conners’ Adult ADHD Rating Scales (CAARS). The DSM-IV ADHD Symptoms Total was analyzed by comparing scores between patients and observers. The significance level was set at 0.10 for exploratory purposes. Statistical analyses included the Mann-Whitney U test and Kruskal-Wallis test to explore the effects of observer gender and relationship on the discrepancy in ADHD symptom reporting.

**Results:**

1. Gender of the patient did not influence self-reported ADHD scores (*U = 5057.500, p = 0.705)*, but observer gender significantly impacted their rating of the patient’s symptoms (*U = 5312.500, p = 0.032).*
2. Female patients’ ADHD symptoms were underrepresented more than male patients’ (U = 3941.500, p = 0.019).
3. Male observers underreported symptoms more than female observers (U = 4772.500, p = 0.075).
4. Observer relationship to the patient did not significantly affect ADHD symptom score discrepancies (H(3) = 4.928, p = 0.177).
5. Female partners were less likely to underrepresent ADHD symptoms compared to female parents, female and male family members, and male partners *(p = 0.028, 0.002, <0.001).*

**Conclusions:**

Female patients were more likely to have their ADHD symptoms underrepresented, particularly by male observers and male partners, indicating potential gender biases in perception. The findings suggest that clinicians should be cautious of possible underreporting of ADHD symptoms by male observers and particularly male partners. Future research should explore whether hyperactivity or inattentiveness is more frequently underrepresented and further validate these exploratory findings.

**Disclosure of Interest:**

None Declared

